# Treatment of Phthisis at High Altitudes

**Published:** 1889-01-19

**Authors:** 


### TREATMENT OF PHTHISIS AT HIGH ALTITUDES.

In a recent number of the *Lancet* Sir Andrew Clark called attention to an important practical point in connexion with the treatment of phthisis at high altitudes. It is not a point which the general reader will appreciate, but the practising physician will do well to note and remember it for the remainder of his life. Sir Andrew expresses his decided conviction that consumptives, as a rule, are well advised when they seek dry, mountainous regions. This is by no means a new idea. The late Sir Robert Christison, who had few equals either in his own or any other generation, was constantly in the habit of inpressing upon his students the value of the pure and fine air which is to be found in largest abundance in certains mountainous regions. So far, therefore, as commending these is concerned, Sir Andrew is saying nothing but what is known to most rational and "non-faddy" physicians. The point, however, to which he calls particular attention, and which he emphasises in the way so characteristic of him, is, so far as we are aware, all his own. In all cases of phthisis, says Sir Andrew, albuminuria may supervene. This is common knowledge. But its probability is determined by the character of the phthisis. In "tubercular" consumption it appears seldomest and latest, and exerts the smallest influence upon the history of the case. In "pneumonic" cases the probability is greater, and the influence also greater; in "Fibroid" phthisis both the probability is greatest and also the intensity and the consequences. The very apex, however, of Sir Andrew's point is this, that cases of phthisis, complicated by the presence of albuminuria, do not do well in the cold. The high frigid regions to which consumptives are sent may suit the lungs, but do not suit the kidneys whenever albuminuria is present. Not only does the temperature exert an evil influence, but a sudden chill may produce a rapidly-fatal result. All medical men who purpose sending patients to high altitudes should carefully examine the urine every day or two for some weeks beforehand. If albumen appears the patient had better, Sir Andrew Clark thinks, either stay at home or seek a milder climate than that of the mountains. If, after he has gone to the hills, albuminuria should appear, and be at all marked or frequent, he had better at once proceed home. Practical points like this really advance the science as well as the art of medicine, and should be recorded everywhere; most of all in the mind of the practising physician.

